# Tubal ectopic pregnancy: comparative management between pre and Covid-19 pandemic periods

**DOI:** 10.61622/rbgo/2024rbgo64

**Published:** 2024-05-27

**Authors:** Lumi Tomishige Chaves, Rafael Maia, Alberto Borges Peixoto, Edward Araujo Júnior, Júlio Elito

**Affiliations:** 1 Universidade Federal de São Paulo Escola Paulista de Medicina Department of Obstetrics São Paulo SP Brazil Department of Obstetrics, Escola Paulista de Medicina, Universidade Federal de São Paulo, São Paulo, SP, Brazil.; 2 Universidade de Uberaba Mário Palmério University Hospital Gynecology and Obstetrics Service Uberaba MG Brazil Gynecology and Obstetrics Service, Mário Palmério University Hospital, Universidade de Uberaba, Uberaba, MG, Brazil.; 3 Universidade Federal do Triângulo Mineiro Department of Obstetrics and Gynecology Uberaba MG Brazil Department of Obstetrics and Gynecology, Universidade Federal do Triângulo Mineiro, Uberaba, MG, Brazil.

**Keywords:** Tubal ectopic pregnancy, Pandemics, Risk factors

## Abstract

**Objective:**

To evaluate whether there were differences in the presentation of patients with tubal ectopic pregnancy (EP) during the first year of the COVID-19 pandemic.

**Methods:**

We performed a retrospective cohort study of all cases of tubal EP between March 2019 and March 2020 (pre-pandemic) and between March 2020 and March 2021 (pandemic). We compared between these two groups the risk factors, clinical characteristics, laboratory data, sonographic aspects, treatment applied and complications.

**Results:**

We had 150 EP diagnoses during the two years studied, of which 135 were tubal EP. Of these, 65 were included in the pre-pandemic and 70 in the pandemic period. The prevalence of lower abdominal pain was significantly higher in the pandemic compared to the pre-pandemic period (91.4% vs. 78.1%, p=0.031). There was no significant difference in shock index, initial beta-hCG level, hemoglobin level at diagnosis, days of menstrual delay, aspect of the adnexal mass, amount of free fluid on ultrasound, and intact or ruptured presentation between the groups. Expectant management was significantly higher during the pandemic period (40.0% vs. 18.5%, p=0.008), surgical management was lower during the pandemic period (47.1% vs. 67.7%, p=0.023), and number of days hospitalized was lower in the pandemic period (1.3 vs. 2.0 days, p=0.003).

**Conclusion:**

We did not observe a significant difference in patient history, laboratory and ultrasound characteristics. Abdominal pain was more common during the pandemic period. Regarding treatment, we observed a significant increase in expectant and a decrease in surgical cases during the pandemic period.

## Introduction

The year 2020 was marked globally by the new coronavirus. At the end of February, the first case of coronavirus disease (COVID-19) was confirmed in Brazil, and in March, the World Health Organization declared a pandemic. To stop the spread of the virus, several countries around the world declared social distancing and strict isolation, including some states in Brazil, such as São Paulo. Then, during this first year of the pandemic, we saw drastic behavioral and psychological changes worldwide, as well as transformations in various sectors of society, especially in the health system. The reduction of services considered non-essential, the instruction to stay at home, and the fear of exposure to the new virus led to a drastic decrease not only in elective surgeries and medical appointments, but also in emergency room visits.^1–4^ This environment may have led to delayed diagnosis of a variety of diseases and conditions, including ectopic pregnancy (EP).^5^

Ectopic pregnancy occurs when the embryo implants and develops outside the uterus. It is the leading cause of maternal death in the first trimester, accounting for 4% of pregnancy-related deaths.^6,7^ Early diagnosis is important because it reduces the risk of tubal rupture and allows for more conservative treatment.^8^ Ectopic pregnancy can be managed surgically, medically, or expectantly.

During the pandemic, some guidelines have been proposed for the management of patients with EP.^9–12^ These guidelines emphasize the importance of early diagnosis in patients with risk factors. Once the diagnosis of EP is confirmed, the guidelines recommend conservative outpatient management (methotrexate or expectant) in appropriate cases to avoid hospitalization. Some studies have shown an increase in tubal rupture cases during the pandemic period,^4,13^ while other authors have not observed this difference.^14^

Therefore, the aim of this study was to evaluate whether there was an impact on the time of diagnosis and, consequently, on the clinical presentation, management and prognosis of patients with EP during the first year of the pandemic compared to the pre-pandemic period.

## Methods

We performed a retrospective cohort study of all cases of EPs diagnosed and followed-up at São Paulo Hospital between March 24, 2019 and March 23, 2020 (pre pandemic period), and March 24, 2020 and March 23, 2021 (first year of the pandemic). The study was approved by the Ethics Committee of Federal University of São Paulo (registration number 59249422.4.0000.5505). We then collected data from all these 150 patients using the inclusion criteria, all EPs, and exclusion criteria, non-tubal EPs. We analyzed age, marital status, date of diagnosis, menstrual delay, number of pregnancies, parity, number of miscarriages, risk factors, vital signs, beta-hCG and hemoglobin levels at diagnosis, clinical presentation, ultrasound characteristics, failure or success of conservative treatment, and final management. Our service follows the most recent guidelines available in the literature for the management of ectopic pregnancy.^15^ Expectant management may be indicated in hemodynamically stable patients with a decline in beta-hCG levels in the range of 24 to 48 hours without treatment, initial beta-hCG levels < 2,000 mIU/mL, no fetal heart activity, and tubal mass < 5.0 cm. Medical treatment with systemic methotrexate is used in hemodynamically stable patients with an adnexal mass up to 3.5 cm, no fetal heart activity, absence of severe or persistent abdominal pain, initial beta-hCG levels < 5,000 mIU/mL, and a rise in beta-hCG levels in the 24 to 48 hours prior to treatment. It is generally administered in a single-dose protocol by intramuscular injection at a dose of 50 mg/m^2^ of calculated body surface area; however, 14-20% of patients require a repeat dose.^15–17^ Less commonly used, the multi-dose regimen may be appropriate for patients presenting with non-tubal EP and initial beta-hCG levels > 5000 mIU/l. It consists of up to 4 doses of methotrexate intramuscular injection (1 mg/kg) on days 1, 3, 5 and 7 and folinic acid at a dose of 0.1 mg/kg on alternate days.^11,17^ Surgical treatment is indicated in patients with a ruptured EP and in those who do not meet the medical or expectant management conditions described above. After determining which approach to follow in the protocol, the risks and benefits of surgical versus non-surgical management were discussed with the patient. If the patient opted for surgical treatment despite meeting the conservative treatment criteria, we did not impose conservative treatment and therefore submitted the patient to "requested" surgery. Those who were expectant or under medical management were followed on an outpatient basis until beta-hCG levels were negative. Data were collected from the patient's chart, transferred to Excel 2019 spreadsheet (Microsoft Corp., Redmond, WA, USA), and irreversibly anonymized by one of our researchers before being made available to a second researcher, who performed the statistical analysis using the SPSS version 20.0 program (SPSS Inc., Chicago, IL, USA) and Prisma GraphPad version 7.0 (GraphPad Software, San Diego, CA, USA). Quantitative variables were subjected to normality test (D'Agostino- Pearson) and those with normal distribution were presented by means and standard deviations (SD). The variables that showed non-normal distribution were presented by medians and minimum and maximum values. The categories of variables were described by absolute and percentage frequencies and presented in tables. To study the difference between the categorical variables and their proportions, the Chi-square test was used. To study the difference between continuous variables, Student-t test was used for variables with normal distribution and the Mann-Whitney test for variables with non-normal distribution. The level of significance for all tests was p < 0.05.

## Results

Of the total 150 patients, 5 had interstitial, 1 had ovarian EP, and 9 had cesarean scar EP, which were excluded from the study. The tubal EP cases included in the study were divided into two groups: Group 1 (n=70) - diagnosis of EP during the pandemic period and Group 2 (n=65) - diagnosis of EP during the pre-pandemic period. There was no significant association between groups, age of patients (p=0.236), marital status (p=0.250), number of pregnancies (p=0.987), parity (p=0.835) and number of miscarriages (p=0.689) (Table 1).

**Table 1:** Demographic characteristics of the studied population

Demographic characteristics	Pandemic (n=70)	Pre-pandemic (n=65)	p-value
Age (years)	32.0 (19.0-44.0)	29.0 (16.0-40.0)	0.236 †
Marital status			0.250 §
Married	16.9% (11/65)	26.2% (17/65)	
Stable union	26.2% (17/65)	30.8% (20/65)	
Single	56.9% (37/65)	43.1% (28.65)	
Pregnancies	2.0 (1.0-6.0)	2.0 (1.0-9.0)	0.987 †
Parity	1.0 (0.0-4.0)	0.0 (0.0-6.0)	0.837 †
Miscarriages	0.0 (0.0-3.0)	0.0 (0.0-5.0)	0.689 †

Mann Whitney

† median (minimum-maximum); Chi-square

§ percentage (n/N). p<0.05

When analyzing the main risk factors for EP, there was no significant association between the studied groups and the presence of previous EP (p=0.242), history of previous cesarean section (p=0.117), smoking (p=0.499), endometriosis (p=0.507), and previous pelvic surgery (p=0.679). There were no patients with previous pelvic inflammatory disease and in vitro fertilization (Table 2).

**Table 2:** Association between the period of pandemic and pre-pandemic and main risk factors for ectopic pregnancy

Risk factors	Pandemic (n=70)	Pre-pandemic (n=65)	p-value §
Previous ectopic pregnancy	20.0% (14/70)	12.5% (8/64)	0.242
Previous cesarean	14.3% (10/70)	25.0% (16/64)	0.117
PID	0.0% (0/70)	0.0% (0/65)	*
Smoking	11.4% (8/70)	15.4% (10/65)	0.499
IVF	0.0% (0/70)	0.0% (0/65)	*
Emergency contraception	2.9% (2/70)	3.1% (2/64)	0.927
Endometriosis	1.4% (1/70)	3.1% (2/64)	0.507
Previous pelvic surgery	40.0% (28/70)	36.5% (23/63)	0.679

PID: pelvic inflammatory disease; IVF: in vitro fertilization. Chi-Square

§ percentage (n/N),

* it was not possible to apply statistical tests due to less than two cases in each group. p<0.05

At the time of diagnosis, a significant association was observed between the study groups and a history of lower abdominal pain (p=0.031), which was significantly higher in the pandemic period compared to the pre-pandemic period (91.4% vs. 78.1%, p=0.031). The risk of a patient diagnosed with EP reporting lower abdominal pain at diagnosis was 2.98 times higher in the pandemic period compared to the pre-pandemic period (OR: 2.98, CI95% 1.11 - 8.27). There was no significant association between the compared groups considering the variables first medical visit (i.e., if the patient had been to other health services before arriving at our hospital) (p=0.574), history of bleeding (p=0.628), shock index (p=0.320), initial dosage of beta-hCG (p=0.052), hemoglobin level at diagnosis (p=0.068) and number of days of menstrual delay (p=0.482) (Table 3).

**Table 3:** Comparison between main clinical and laboratory data at the time of initial care and diagnosis of ectopic pregnancy in the pandemic and pre-pandemic periods

Clinical data	Pandemic (n=70)	Pre-pandemic (n=65)	p-value
First medical visit	32.9% (23/70)	37.5% (24/64)	0.574 §
SI (HR/SBP)	0.74 (0.15)	0.76 (0.13)	0.320 ∫
Beta-hCG diagnosis level (mUI/ml)	1601.0 (25.9-37795.0)	2998.0 (39.6-89368)	0.052 †
Hb level at diagnosis (g/dl)	12.3 (6.60-15.1)	12.9 (9.0-14.5)	0.068 †
Menstrual delay (days)	52.0 (34.0-87.0)	52.0 (33.0-73.0)	0.482 †
Bleeding	94.3% (66/70)	92.2 % (59/64)	0.628 §
LA pain	91.4% (64/70)	78.1% (50/64)	0.031 §

SI: shock index; HR: heart rate; SBP: systolic blood pressure; hCG: human chorionic gonadotropin; mUI: milli-international units, Hb: hemoglobin; LA: lower abdomen. Student-t test

∫ mean (standard deviation); Mann Whitney

† median (quartile); Chi-square

§ percentage (n/N). p<0.05

No significant association was observed between the aspect of the adnexal mass (p=0.117), the amount of free fluid (p=0.755), and the diameter of the adnexal mass (p=0.478) during the pandemic and pre-pandemic periods (Table 4).

**Table 4:** Comparison between the sonographic characteristics during the pandemic and pre-pandemic periods

Sonographic characteristics	Pandemic (n=70)	Pre-pandemic (n=65)	p-value
Aspect			0.117 §
Tubal ring	80.0% (56/70)	75.4% (49/65)	0.514 §
Hematosalpinx	1.4% (1/70)	3.1% (2/65)	0.608 §
Gestational sac	4.3% (3/70)	0.0% (0/65)	0.245 §
Embryo with heart activity	10.0% (7/70)	20.0% (13/65)	0.145 §
Embryo without heart activity	4.3% (3/70)	1.5% (1/65)	0.620 §
Free fluid			0.755 §
Absent	41.4% (29/70)	40.0% (26/65)	> 0.999 §
Low	30.0% (21/70)	29.2% (19/65)	> 0.999 §
Mild	24.3% (17/70)	29.2% (19/65)	0.562 §
Large	4.3% (3/70)	1.5% (1/65)	0.620 §
Diameter (cm)	3.0 (1.0-12.1)	2.8 (1.2-10.4)	0.478 †

Mann Whitney

† median (quartile); Chi-Square

§ percentage (n/N). p<0.05

Regarding the evolution of cases, no significant association was observed between the prevalence of unruptured and ruptured EP in the pandemic or pre-pandemic periods (p=0.155). During the pandemic period, 47.1%, 40.0%, and 12.9% of cases were managed by surgery, expectant management, and clinical management, respectively. When comparing the association of the study period with the final approach adopted, it was observed that the use of expectant management for EP was significantly higher (40.0% vs. 18.5%, p=0.008), while the use of surgical treatment was significantly lower (47.1% vs. 67.7%, p=0.023) in the pandemic compared to the pre-pandemic period. A significantly lower mean number of days of hospitalization was observed in the pandemic compared to the pre-pandemic period (1.3 vs. 2.0 days, p=0.003). No significant difference was observed between the type of clinical treatment with methotrexate and the type of surgical treatment in the two periods. No significant association was observed between the pandemic and pre-pandemic periods and failure of conservative treatment (p=0.676) (Table 5).

**Table 5:** Evolution of cases during the pandemic and pre-pandemic periods

Evolution	Pandemic (n=70)	Pre-pandemic (n=65)	p-value
Presentation			
Unruptured	68.6% (48/70)	55.4% (36/65)	0.155 §
Ruptured	31.4% (22/65)	44.6% (29/65)	0.155 §
Final treatment			
Expectant	40.0% (28/70)	18.5% (12/65)	0.008 §
Methotrexate unique dose	10.0% (7/70)	12.3% (8/65)	0.786 §
Methotrexate multiple dose	2.9% (2/70)	1.5% (1/65)	>0.999 §
Surgery	47.1% (33/70)	67.7% (44/65)	0.023 §
Urgence	69.7% (23/33)	63.6% (28/44)	0.632 §
Elective	30.3% (10/33)	36.4% (16/44)	0.632 §
Laparoscopy	12.1% (4/33)	22.7% (10/44)	0.371 §
Laparotomy	87.9% (29/33)	77.3% (34/44)	0.371 §
Hospitalization (days)	1.3 (1.2)	2.0 (1.2)	0.003 ∫
Failure of conservative treatment	11.4% (8/70)	9.2% (6/65)	0.676 §
Expectant	25% (2/8)	33% (2/6)	>0.999 §
Methotrexate	75% (6/8)	66% (4/6)	>0.999 §

Student-t test

∫ mean (standard deviation); Chi-square

§ percentage (n/N). p<0.05

## Discussion

In this study, during the pandemic period, lower abdominal pain was the complaint that most motivated hospital visits. This may reflect the population's fear of leaving their isolation and only going to the hospital when faced with a symptom that could not be postponed. In these cases, it is possible that some of these patients waited longer at home before seeking medical care, whereas they would have sought care earlier had they not been in the pandemic period.

Regarding treatment, our initial hypothesis was consistent with the majority of the available literature on the impact of the COVID-19 pandemic on EP management, which showed higher rates of tubal EP rupture and subsequent surgical treatment of patients.^4,18–21^ There are other studies showing no statistical difference in the outcome and treatment choice of EP between these two periods.^14,22,23^ However, in our study, the percentage of patients who underwent surgery during the pandemic period was significantly lower, while the percentage of patients who were managed expectantly was significantly higher. To our knowledge, there is only one other study with similar results to ours, a prospective study conducted in the United Kingdom, which showed a lower percentage of patients treated surgically and an increase in non-surgical treatment (methotrexate and expectant management combined).^24^

Based on our results, we speculated on the factors that may have influenced and led to this statistical difference. When the diagnosis of an unruptured EP is confirmed, based on the level of beta-hCG, the size of the mass and its appearance on ultrasound, the physician selects the most appropriate treatment. We must take into account that in our institution, as mentioned above, we offer the patient the possibility of choosing between conservative treatment, if applicable, or direct surgical treatment, depending on the case. We believe that before the pandemic, some patients in this type of case would have opted for a faster and more definitive surgical treatment. However, during the pandemic period, the majority of patients most likely prioritized avoiding hospitalization to reduce their exposure to the virus, and therefore chose non-surgical treatment.

Our study has remarkable statistical value because we had a relatively large sample of patients and none of them were left behind, since we manually checked every appointment during these two years. In addition, all patients, without exception, were followed from diagnosis to the end of treatment.

## Conclusion

In summary, we observed that the complaint of abdominal pain was more common during the pandemic period. There was significant increase in expectant management and a decrease in surgical cases during the pandemic period. It is possible that patients arrived at the hospital when the risk of tubal rupture had passed. Another explanation is that patients and physicians were more inclined to avoid hospitalization and opt for non-surgical approaches if they met the inclusion criteria.
